# 4-[(*Z*)-Allyl­amino­(phen­yl)methyl­ene]-3-methyl-1-phenyl-1*H*-pyrazol-5(4*H*)-one

**DOI:** 10.1107/S160053681001384X

**Published:** 2010-05-12

**Authors:** Hai-Zhen Xu, Yan-Xia Yang, Jing Yan, You-Quan Zhu

**Affiliations:** a College of Chemistry, Tianjin Normal University, 393 Binshuixi Road, Xiqing District, Tianjin 300387, People’s Republic of China; b Nankai High School, 100 Sima Road, Nankai District, Tianjin 300100, People’s Republic of China; c State Key Laboratory of Elemento-Organic Chemistry, Nankai University, Tianjin 300071, People’s Republic of China

## Abstract

The title compound, C_20_H_19_N_3_O, exists in a keto–enamine tautomeric form. The pyrazolone ring makes dihedral angles of 20.52 (10) and 77.73 (5)° with the two phenyl rings and an intra­molecular N—H⋯O hydrogen bond occurs. A weak inter­molecular C—H⋯O hydrogen bond is observed in the crystal structure. The allyl group is disordered over two positions, with site-occupancy factors of 0.533 (5) and 0.467 (5).

## Related literature

For the analgesic activity of metal complexes with 1-phenyl-3-methyl-4-benzoyl­pyrazolon-5-one, see: Li *et al.* (1997 ▶); Liu *et al.* (1980 ▶); Zhou *et al.* (1999 ▶). For related structures, see: Bao *et al.* (2004 ▶); Sun *et al.* (2007 ▶); Zhu *et al.* (2005 ▶).
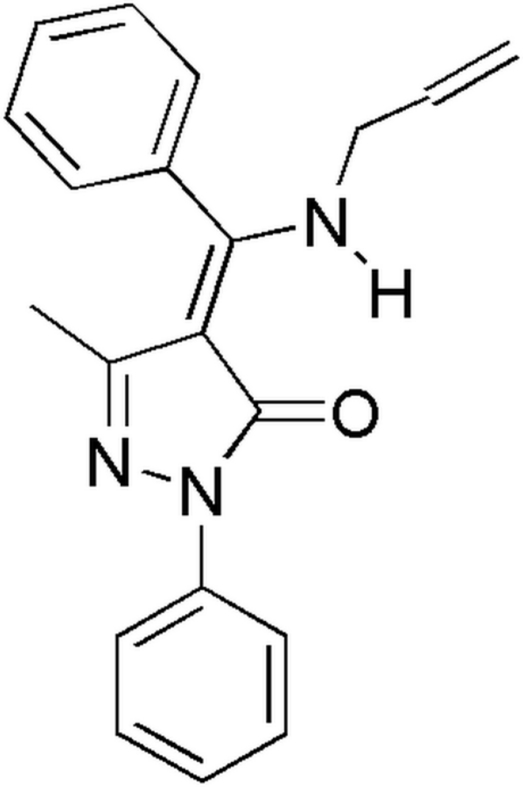


## Experimental

### 

#### Crystal data

C_20_H_19_N_3_O
*M* *_r_* = 317.38
Triclinic, 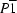

*a* = 9.295 (1) Å
*b* = 9.8440 (12) Å
*c* = 10.0670 (14) Å
α = 86.175 (8)°
β = 89.280 (9)°
γ = 74.329 (7)°
*V* = 884.90 (19) Å^3^
*Z* = 2
Mo *K*α radiation
μ = 0.08 mm^−1^
*T* = 293 K
0.22 × 0.18 × 0.16 mm

#### Data collection

Rigaku Saturn724 CCD camera diffractometer
Absorption correction: multi-scan (*CrystalClear*; Rigaku, 2009 ▶) *T*_min_ = 0.984, *T*_max_ = 0.988
10726 measured reflections
3910 independent reflections
2135 reflections with *I* > 2σ(*I*)
*R*_int_ = 0.032

#### Refinement

*R*[*F* ^2^ > 2σ(*F* ^2^)] = 0.047
*wR*(*F* ^2^) = 0.133
*S* = 0.94
3910 reflections
242 parameters
16 restraints
H atoms treated by a mixture of independent and constrained refinement
Δρ_max_ = 0.17 e Å^−3^
Δρ_min_ = −0.19 e Å^−3^

### 

Data collection: *CrystalClear* (Rigaku, 2009 ▶); cell refinement: *CrystalClear*; data reduction: *CrystalClear*; program(s) used to solve structure: *SHELXS97* (Sheldrick, 2008 ▶); program(s) used to refine structure: *SHELXL97* (Sheldrick, 2008 ▶); molecular graphics: *CrystalStructure* (Rigaku, 2009 ▶); software used to prepare material for publication: *CrystalStructure*.

## Supplementary Material

Crystal structure: contains datablocks global, I. DOI: 10.1107/S160053681001384X/is2538sup1.cif

Structure factors: contains datablocks I. DOI: 10.1107/S160053681001384X/is2538Isup2.hkl

Additional supplementary materials:  crystallographic information; 3D view; checkCIF report

## Figures and Tables

**Table 1:** Hydrogen-bond geometry (Å, °)

*D*—H⋯*A*	*D*—H	H⋯*A*	*D*⋯*A*	*D*—H⋯*A*
N3—H3⋯O1	1.06 (2)	1.75 (2)	2.687 (2)	145 (2)
C17—H17⋯O1^i^	0.95	2.41	3.345 (2)	168

Symmetry code: (i) 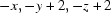.

**Fig. 1.**
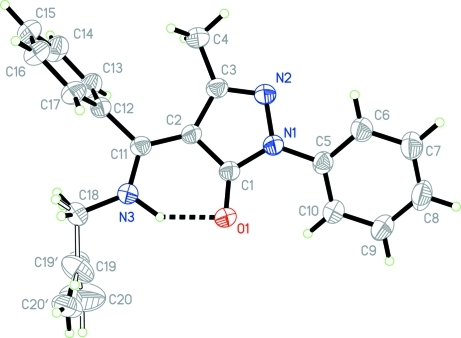
View of the title compound, with displacement ellipsoids drawn at the 50% probability level.

**Fig. 2.**
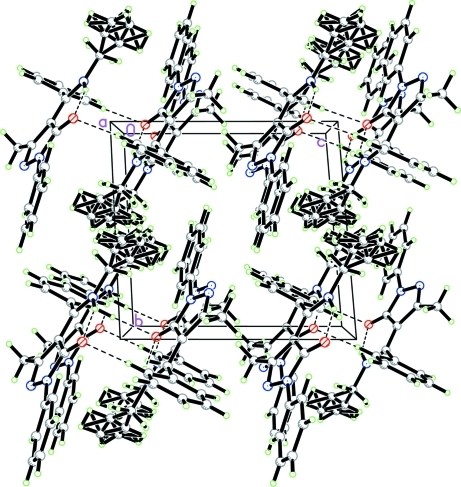
Intermolecular hydrogen bonds (dashed line) in the structure of (I).

## supplementary crystallographic information

### Comment

1-Phenyl-3-methyl-4-benzoylpyrazolon-5-one (HPMBP), an effective β-diketonate, is widely used and well known for its extractive ability. In recent years, HPMBP and its metal complexes have also been found to have good antibacterial and biological properties. Its metal complexes have analgesic activity (Liu *et al.*, 1980; Li *et al.*, 1997; Zhou *et al.*, 1999). In order to develop new medicines, we have synthesized the title compound, (I), and its structure is reported here.

The structure of (I) is shown in Fig. 1. The dihedral angles formed by the pyrazolone ring with the two phenyl rings C5–C10 and C12–C17 are 20.52 (10) and 77.73 (5)°, respectively. The O atom of the 3-methyl-1-phenylpyrazol-5-one moiety and the N atom of the allylamino group are available for coordination with metals. The pyrazole ring is planar and atoms O1, C1, C2, C11 and N3 are almost coplanar, the largest deviation being 0.0195 (11) Å for atom C11. The dihedral angle between this mean plane and the pyrazoline ring of PMBP is 2.01 (12)°. The bond lengths within this part of the molecule lie between classical single- and double-bond lengths, indicating extensive conjugation. A strong intramolecular N3—H3···O1 hydrogen bond (Table 1) is observed, leading to a keto-enamine form. The crystal structure includes intermolecular C—H···O hydrogen bonds (Table 1 and Fig. 2).

### Experimental

Compound (I) was synthesized by refluxing a mixture of 1-phenyl-3-methyl-4-benzoylpyrazol-5-one (10 mmol) and allylamine (10 mmol) in ethanol (80 ml) over a steam bath for about 10 h. Excess solvent was removed by evaporation and the solution was cooled to room temperature. After 4 d, a colorless solid was obtained and this was dried in air. The product was recrystallized from ethanol, to afford colorless crystals of (I) suitable for X-ray analysis.

### Refinement

C-bound H atoms were positioned geometrically, with C—H = 0.95–0.96 Å and were refined as riding, with *U*_iso_(H) = 1.2*U*_eq_(C). The amine H atom (H3) found in a difference map was refined freely. The allyl group shows positional disorder. In the final refinement, the occupancy factors of two possible sites, C19/C20 and C19'/C20', converged to 0.533 (5) and 0.467 (5). For the disordered unit, distance restraints [C18—C19 = C18—C19' = 1.50 (1) Å and C19—C20 = C19'—C20' = 1.34 (1) Å] were applied. The terminal C20 and C20' atoms were also restrained to be approximately isotropic (*ISOR*).

### Crystal data

**Table:**

C_20_H_19_N_3_O	*Z* = 2
*M**_r_* = 317.38	*F*(000) = 336
Triclinic, *P*1	*D*_x_ = 1.191 Mg m^−^^3^
Hall symbol: -P 1	Mo *K*α radiation, λ = 0.71075 Å
*a* = 9.295 (1) Å	Cell parameters from 2516 reflections
*b* = 9.8440 (12) Å	θ = 2.0–27.2°
*c* = 10.0670 (14) Å	µ = 0.08 mm^−^^1^
α = 86.175 (8)°	*T* = 293 K
β = 89.280 (9)°	Prism, colorless
γ = 74.329 (7)°	0.22 × 0.18 × 0.16 mm
*V* = 884.90 (19) Å^3^	

### Data collection

**Table:**

Rigaku Saturn724 CCD camera diffractometer	3910 independent reflections
Radiation source: rotating anode	2135 reflections with *I* > 2σ(*I*)
multilayer	*R*_int_ = 0.032
ω scans	θ_max_ = 27.2°, θ_min_ = 2.0°
Absorption correction: multi-scan (*CrystalClear*; Rigaku, 2009)	*h* = −11→11
*T*_min_ = 0.984, *T*_max_ = 0.988	*k* = −12→12
10726 measured reflections	*l* = −12→12

### Refinement

**Table:**

Refinement on *F*^2^	Secondary atom site location: difference Fourier map
Least-squares matrix: full	Hydrogen site location: inferred from neighbouring sites
*R*[*F*^2^ > 2σ(*F*^2^)] = 0.047	H atoms treated by a mixture of independent and constrained refinement
*wR*(*F*^2^) = 0.133	*w* = 1/[σ^2^(*F*_o_^2^) + (0.0682*P*)^2^] where *P* = (*F*_o_^2^ + 2*F*_c_^2^)/3
*S* = 0.94	(Δ/σ)_max_ < 0.001
3910 reflections	Δρ_max_ = 0.17 e Å^−^^3^
242 parameters	Δρ_min_ = −0.19 e Å^−^^3^
16 restraints	Extinction correction: *SHELXL97* (Sheldrick, 2008), Fc^*^=kFc[1+0.001xFc^2^λ^3^/sin(2θ)]^-1/4^
Primary atom site location: structure-invariant direct methods	Extinction coefficient: 0.044 (6)

### Special details

**Table:**

Geometry. All esds (except the esd in the dihedral angle between two l.s. planes) are estimated using the full covariance matrix. The cell esds are taken into account individually in the estimation of esds in distances, angles and torsion angles; correlations between esds in cell parameters are only used when they are defined by crystal symmetry. An approximate (isotropic) treatment of cell esds is used for estimating esds involving l.s. planes.
Refinement. Refinement of *F*^2^ against ALL reflections. The weighted *R*-factor wR and goodness of fit *S* are based on *F*^2^, conventional *R*-factors *R* are based on *F*, with *F* set to zero for negative *F*^2^. The threshold expression of *F*^2^ > σ(*F*^2^) is used only for calculating *R*-factors(gt) etc. and is not relevant to the choice of reflections for refinement. *R*-factors based on *F*^2^ are statistically about twice as large as those based on *F*, and *R*- factors based on ALL data will be even larger.

### Fractional atomic coordinates and isotropic or equivalent isotropic displacement parameters (Å^2^)

**Table:**

	*x*	*y*	*z*	*U*_iso_*/*U*_eq_	Occ. (<1)
O1	0.20365 (11)	0.99841 (12)	0.85524 (11)	0.0659 (4)	
N1	0.08977 (14)	1.17446 (14)	0.69281 (13)	0.0581 (4)	
N2	−0.03759 (15)	1.20734 (14)	0.61172 (14)	0.0623 (4)	
N3	0.04161 (16)	0.81085 (15)	0.89396 (14)	0.0668 (4)	
H3	0.130 (2)	0.858 (2)	0.9078 (19)	0.098 (6)*	
C1	0.10280 (17)	1.05164 (17)	0.77140 (15)	0.0532 (4)	
C2	−0.02439 (16)	1.00449 (16)	0.73734 (15)	0.0523 (4)	
C3	−0.10420 (17)	1.10712 (17)	0.63795 (16)	0.0570 (4)	
C4	−0.2423 (2)	1.1118 (2)	0.56172 (19)	0.0750 (5)	
H4A	−0.2771	1.2037	0.5118	0.090*	
H4B	−0.3202	1.0983	0.6237	0.090*	
H4C	−0.2203	1.0364	0.4996	0.090*	
C5	0.17910 (19)	1.27022 (17)	0.69264 (16)	0.0593 (4)	
C6	0.1197 (2)	1.40867 (19)	0.64376 (18)	0.0724 (5)	
H6	0.0188	1.4392	0.6140	0.087*	
C7	0.2068 (3)	1.5026 (2)	0.6381 (2)	0.0905 (7)	
H7	0.1662	1.5972	0.6031	0.109*	
C8	0.3519 (3)	1.4595 (2)	0.6831 (2)	0.0945 (7)	
H8	0.4120	1.5238	0.6788	0.113*	
C9	0.4100 (2)	1.3225 (2)	0.7344 (2)	0.0852 (6)	
H9	0.5097	1.2934	0.7674	0.102*	
C10	0.3249 (2)	1.2272 (2)	0.73843 (19)	0.0716 (5)	
H10	0.3664	1.1325	0.7725	0.086*	
C11	−0.05472 (16)	0.88577 (16)	0.80315 (15)	0.0531 (4)	
C12	−0.19394 (17)	0.84403 (16)	0.77922 (16)	0.0536 (4)	
C13	−0.2147 (2)	0.77965 (19)	0.66608 (18)	0.0703 (5)	
H13	−0.1366	0.7554	0.6029	0.084*	
C14	−0.3499 (2)	0.7506 (2)	0.6453 (2)	0.0828 (6)	
H14	−0.3645	0.7065	0.5674	0.099*	
C15	−0.4624 (2)	0.7848 (2)	0.7356 (2)	0.0779 (6)	
H15	−0.5556	0.7662	0.7197	0.093*	
C16	−0.44114 (19)	0.8458 (2)	0.8489 (2)	0.0732 (5)	
H16	−0.5192	0.8681	0.9122	0.088*	
C17	−0.30750 (18)	0.87516 (18)	0.87209 (17)	0.0642 (5)	
H17	−0.2931	0.9167	0.9516	0.077*	
C18	0.0316 (2)	0.68276 (19)	0.9706 (2)	0.0775 (6)	
H18A	−0.0194	0.6319	0.9183	0.093*	0.533 (9)
H18B	−0.0251	0.7075	1.0499	0.093*	0.533 (9)
H18C	0.0338	0.6098	0.9113	0.093*	0.467 (9)
H18D	−0.0616	0.7019	1.0174	0.093*	0.467 (9)
C19	0.1772 (5)	0.5941 (6)	1.0070 (7)	0.0726 (16)	0.533 (9)
H19	0.2349	0.5531	0.9340	0.087*	0.533 (9)
C20	0.2430 (9)	0.5605 (8)	1.1187 (6)	0.106 (2)	0.533 (9)
H20A	0.1939	0.5966	1.1973	0.128*	0.533 (9)
H20B	0.3415	0.4993	1.1237	0.128*	0.533 (9)
C19'	0.1553 (8)	0.6368 (9)	1.0669 (8)	0.098 (2)	0.467 (9)
H19'	0.1399	0.6733	1.1525	0.118*	0.467 (9)
C20'	0.2789 (10)	0.5535 (13)	1.0447 (12)	0.178 (4)	0.467 (9)
H20C	0.2984	0.5148	0.9602	0.214*	0.467 (9)
H20D	0.3537	0.5286	1.1122	0.214*	0.467 (9)

### Atomic displacement parameters (Å^2^)

**Table:**

	*U*^11^	*U*^22^	*U*^33^	*U*^12^	*U*^13^	*U*^23^
O1	0.0607 (7)	0.0729 (8)	0.0680 (8)	−0.0270 (6)	−0.0173 (6)	0.0090 (6)
N1	0.0619 (8)	0.0603 (8)	0.0555 (8)	−0.0236 (7)	−0.0107 (7)	0.0028 (7)
N2	0.0655 (9)	0.0643 (9)	0.0591 (9)	−0.0222 (7)	−0.0147 (7)	0.0036 (7)
N3	0.0652 (9)	0.0676 (9)	0.0717 (10)	−0.0284 (7)	−0.0162 (8)	0.0144 (8)
C1	0.0540 (9)	0.0574 (10)	0.0496 (9)	−0.0171 (7)	−0.0043 (8)	−0.0023 (8)
C2	0.0510 (9)	0.0576 (9)	0.0503 (9)	−0.0184 (7)	−0.0055 (7)	−0.0023 (8)
C3	0.0556 (9)	0.0645 (10)	0.0520 (10)	−0.0176 (8)	−0.0064 (8)	−0.0032 (8)
C4	0.0705 (12)	0.0822 (13)	0.0747 (13)	−0.0268 (10)	−0.0227 (10)	0.0081 (10)
C5	0.0696 (11)	0.0620 (11)	0.0527 (10)	−0.0284 (9)	−0.0022 (8)	−0.0043 (8)
C6	0.0852 (13)	0.0670 (12)	0.0705 (12)	−0.0303 (10)	−0.0113 (10)	0.0000 (9)
C7	0.1128 (17)	0.0687 (13)	0.0989 (17)	−0.0404 (12)	−0.0136 (14)	−0.0006 (11)
C8	0.1163 (18)	0.0822 (15)	0.1049 (18)	−0.0598 (14)	−0.0044 (14)	−0.0092 (13)
C9	0.0827 (13)	0.0890 (15)	0.0965 (16)	−0.0437 (11)	−0.0090 (12)	−0.0093 (12)
C10	0.0711 (12)	0.0700 (12)	0.0800 (13)	−0.0302 (9)	−0.0061 (10)	−0.0027 (10)
C11	0.0518 (9)	0.0589 (10)	0.0496 (9)	−0.0162 (8)	−0.0045 (7)	−0.0047 (8)
C12	0.0554 (9)	0.0572 (9)	0.0509 (9)	−0.0201 (7)	−0.0051 (8)	−0.0015 (7)
C13	0.0753 (12)	0.0874 (13)	0.0586 (11)	−0.0371 (10)	0.0013 (9)	−0.0164 (10)
C14	0.0929 (14)	0.1009 (15)	0.0694 (13)	−0.0478 (12)	−0.0109 (11)	−0.0197 (11)
C15	0.0674 (12)	0.0878 (14)	0.0875 (15)	−0.0360 (10)	−0.0143 (11)	−0.0044 (12)
C16	0.0557 (10)	0.0876 (13)	0.0795 (13)	−0.0234 (9)	−0.0013 (9)	−0.0108 (11)
C17	0.0580 (10)	0.0765 (12)	0.0617 (11)	−0.0218 (8)	−0.0027 (9)	−0.0140 (9)
C18	0.0852 (13)	0.0719 (12)	0.0779 (13)	−0.0302 (10)	−0.0147 (11)	0.0179 (10)
C19	0.052 (3)	0.067 (3)	0.092 (4)	−0.005 (2)	−0.003 (3)	−0.001 (3)
C20	0.095 (4)	0.118 (4)	0.093 (4)	−0.012 (3)	−0.024 (3)	0.029 (3)
C19'	0.123 (6)	0.092 (5)	0.059 (4)	0.006 (4)	0.005 (4)	0.000 (3)
C20'	0.115 (6)	0.273 (9)	0.110 (7)	0.013 (6)	−0.002 (5)	−0.025 (6)

### Geometric parameters (Å, °)

**Table:**

O1—C1	1.2494 (17)	C11—C12	1.487 (2)
N1—C1	1.378 (2)	C12—C13	1.380 (2)
N1—N2	1.3981 (17)	C12—C17	1.386 (2)
N1—C5	1.4145 (19)	C13—C14	1.383 (2)
N2—C3	1.3106 (19)	C13—H13	0.9500
N3—C11	1.3249 (19)	C14—C15	1.364 (3)
N3—C18	1.458 (2)	C14—H14	0.9500
N3—H3	1.062 (19)	C15—C16	1.364 (3)
C1—C2	1.435 (2)	C15—H15	0.9500
C2—C11	1.398 (2)	C16—C17	1.375 (2)
C2—C3	1.432 (2)	C16—H16	0.9500
C3—C4	1.492 (2)	C17—H17	0.9500
C4—H4A	0.9800	C18—C19	1.436 (5)
C4—H4B	0.9800	C18—C19'	1.468 (6)
C4—H4C	0.9800	C18—H18A	0.9601
C5—C10	1.382 (2)	C18—H18B	0.9600
C5—C6	1.384 (2)	C18—H18C	0.9600
C6—C7	1.382 (3)	C18—H18D	0.9600
C6—H6	0.9500	C19—C20	1.267 (7)
C7—C8	1.373 (3)	C19—H19	0.9500
C7—H7	0.9500	C20—H20A	0.9500
C8—C9	1.378 (3)	C20—H20B	0.9500
C8—H8	0.9500	C19'—C20'	1.246 (8)
C9—C10	1.379 (2)	C19'—H19'	0.9500
C9—H9	0.9500	C20'—H20C	0.9500
C10—H10	0.9500	C20'—H20D	0.9500
			
C1—N1—N2	111.74 (12)	C13—C12—C17	119.47 (15)
C1—N1—C5	128.86 (14)	C13—C12—C11	122.02 (15)
N2—N1—C5	119.21 (13)	C17—C12—C11	118.47 (14)
C3—N2—N1	106.53 (13)	C12—C13—C14	119.53 (18)
C11—N3—C18	126.82 (15)	C12—C13—H13	120.2
C11—N3—H3	110.9 (10)	C14—C13—H13	120.2
C18—N3—H3	122.3 (10)	C15—C14—C13	120.61 (18)
O1—C1—N1	125.78 (15)	C15—C14—H14	119.7
O1—C1—C2	129.30 (15)	C13—C14—H14	119.7
N1—C1—C2	104.91 (13)	C16—C15—C14	119.97 (18)
C11—C2—C3	132.68 (15)	C16—C15—H15	120.0
C11—C2—C1	121.72 (14)	C14—C15—H15	120.0
C3—C2—C1	105.45 (13)	C15—C16—C17	120.51 (18)
N2—C3—C2	111.37 (14)	C15—C16—H16	119.7
N2—C3—C4	118.53 (15)	C17—C16—H16	119.7
C2—C3—C4	130.07 (15)	C16—C17—C12	119.87 (16)
C3—C4—H4A	109.5	C16—C17—H17	120.1
C3—C4—H4B	109.5	C12—C17—H17	120.1
H4A—C4—H4B	109.5	C19—C18—N3	111.1 (3)
C3—C4—H4C	109.5	N3—C18—C19'	110.1 (4)
H4A—C4—H4C	109.5	C19—C18—H18A	109.6
H4B—C4—H4C	109.5	N3—C18—H18A	109.1
C10—C5—C6	119.66 (17)	C19—C18—H18B	109.0
C10—C5—N1	121.11 (15)	N3—C18—H18B	109.6
C6—C5—N1	119.23 (16)	H18A—C18—H18B	108.3
C7—C6—C5	120.17 (19)	N3—C18—H18C	109.6
C7—C6—H6	119.9	C19'—C18—H18C	110.4
C5—C6—H6	119.9	N3—C18—H18D	109.1
C8—C7—C6	120.1 (2)	C19'—C18—H18D	109.3
C8—C7—H7	120.0	H18C—C18—H18D	108.3
C6—C7—H7	120.0	C20—C19—C18	131.8 (9)
C7—C8—C9	119.77 (19)	C20—C19—H19	114.1
C7—C8—H8	120.1	C18—C19—H19	114.1
C9—C8—H8	120.1	C19—C20—H20A	120.0
C8—C9—C10	120.6 (2)	C19—C20—H20B	120.0
C8—C9—H9	119.7	H20A—C20—H20B	120.0
C10—C9—H9	119.7	C20'—C19'—C18	124.8 (11)
C9—C10—C5	119.68 (18)	C20'—C19'—H19'	117.6
C9—C10—H10	120.2	C18—C19'—H19'	117.6
C5—C10—H10	120.2	C19'—C20'—H20C	120.0
N3—C11—C2	118.69 (14)	C19'—C20'—H20D	120.0
N3—C11—C12	118.78 (14)	H20C—C20'—H20D	120.0
C2—C11—C12	122.48 (14)		
			
C1—N1—N2—C3	−0.16 (18)	C8—C9—C10—C5	−1.2 (3)
C5—N1—N2—C3	−175.66 (13)	C6—C5—C10—C9	−0.3 (3)
N2—N1—C1—O1	−178.45 (15)	N1—C5—C10—C9	178.70 (16)
C5—N1—C1—O1	−3.5 (3)	C18—N3—C11—C2	−178.50 (16)
N2—N1—C1—C2	0.05 (17)	C18—N3—C11—C12	4.1 (3)
C5—N1—C1—C2	175.00 (15)	C3—C2—C11—N3	−178.88 (16)
O1—C1—C2—C11	2.3 (3)	C1—C2—C11—N3	−3.9 (2)
N1—C1—C2—C11	−176.11 (14)	C3—C2—C11—C12	−1.5 (3)
O1—C1—C2—C3	178.50 (16)	C1—C2—C11—C12	173.46 (14)
N1—C1—C2—C3	0.07 (16)	N3—C11—C12—C13	−106.78 (19)
N1—N2—C3—C2	0.21 (18)	C2—C11—C12—C13	75.9 (2)
N1—N2—C3—C4	−177.88 (14)	N3—C11—C12—C17	75.2 (2)
C11—C2—C3—N2	175.40 (17)	C2—C11—C12—C17	−102.11 (19)
C1—C2—C3—N2	−0.18 (18)	C17—C12—C13—C14	1.7 (3)
C11—C2—C3—C4	−6.8 (3)	C11—C12—C13—C14	−176.21 (16)
C1—C2—C3—C4	177.63 (16)	C12—C13—C14—C15	−0.1 (3)
C1—N1—C5—C10	24.4 (3)	C13—C14—C15—C16	−1.2 (3)
N2—N1—C5—C10	−161.01 (15)	C14—C15—C16—C17	1.0 (3)
C1—N1—C5—C6	−156.64 (16)	C15—C16—C17—C12	0.7 (3)
N2—N1—C5—C6	18.0 (2)	C13—C12—C17—C16	−2.0 (3)
C10—C5—C6—C7	1.4 (3)	C11—C12—C17—C16	176.02 (15)
N1—C5—C6—C7	−177.61 (17)	C11—N3—C18—C19	151.5 (4)
C5—C6—C7—C8	−1.1 (3)	C11—N3—C18—C19'	−176.4 (4)
C6—C7—C8—C9	−0.4 (3)	N3—C18—C19—C20	111.7 (6)
C7—C8—C9—C10	1.5 (3)	N3—C18—C19'—C20'	−90.6 (11)

### Hydrogen-bond geometry (Å, °)

**Table:**

*D*—H···*A*	*D*—H	H···*A*	*D*···*A*	*D*—H···*A*
N3—H3···O1	1.06 (2)	1.75 (2)	2.687 (2)	145 (2)
C17—H17···O1^i^	0.95	2.41	3.345 (2)	168

Symmetry codes: (i) −*x*, −*y*+2, −*z*+2.
